# Dynamic changes in physical function during intensive chemotherapy affect transplant outcomes in older adults with AML

**DOI:** 10.3389/fonc.2023.1281782

**Published:** 2023-11-07

**Authors:** Gi-June Min, Byung-Sik Cho, Daehun Kwag, Sung-Soo Park, Silvia Park, Jae-Ho Yoon, Sung-Eun Lee, Ki-Seong Eom, Yoo-Jin Kim, Seok Lee, Chang-Ki Min, Seok-Goo Cho, Jong Wook Lee, Hee-Je Kim

**Affiliations:** ^1^ Department of Hematology, Catholic Hematology Hospital, Seoul St. Mary’s Hospital, College of Medicine, The Catholic University of Korea, Seoul, Republic of Korea; ^2^ Leukemia Research Institute, College of Medicine, The Catholic University of Korea, Seoul, Republic of Korea

**Keywords:** geriatric assessment, allogeneic transplantation, acute myeloid leukemia, survival outcomes, physical function

## Abstract

**Introduction:**

Intensive chemotherapy (IC) can affect all geriatric assessment (GA) domains in older adults with acute myeloid leukemia (AML), but data on the effects of these changes on transplant outcomes are lacking.

**Methods:**

Therefore, we prospectively assessed the prognostic role of GA domains at diagnosis and allogeneic hematopoietic stem cell transplantation (allo-HSCT) in 51 patients with AML aged ≥60 years who achieved complete remission after IC. We performed both baseline and pre-allo-HSCT GA; moreover, physical function, including a short physical performance battery (SPPB), cognitive function, psychological function, nutritional status, and social support were examined.

**Results:**

All GA domains showed dynamic changes between the two time points. The directions of change were statistically significant for social support, self-reported physical and psychological functions, and distress, but not for nutritional status, cognitive function, or physical function. Among all GA domains at each time point, only poor physical function and its submaneuvers at diagnosis but not at allo-HSCT were significantly associated with inferior survival. In particular, since the direction of change varied between patients, we found that patients whose physical function improved before allo-HSCT were more likely to survive longer than those with persistently impaired SPPB (55.6% vs. 28.6%, p=0.268). Finally, persistent impairment in SPPB (28.6% vs. 65.9%, p=0.006), tandem stand (0% vs. 63.3%, p=0.012), sit-and-stand (41.2% vs. 70.6%, p=0.009), and gait speed (38.5% vs. 68.4%, p=0.027) further strongly predicted inferior survival.

**Discussion:**

This study showed that IC courses can induce dynamic changes in different directions in the GA domains of each patient and that changes in objectively measured physical function can predict transplant outcomes.

## 1 Introduction

Allogeneic hematopoietic stem cell transplantation (allo-HSCT) remains a curative therapeutic approach for high-risk acute myeloid leukemia (AML); however, it is associated with high morbidity and mortality, particularly in older adults (1). However, the introduction of advanced supportive care and reduced-intensity conditioning has broadened access to allo-HSCT in older patients by reducing toxicity (2, 3). Older adults with AML are increasingly undergoing allo-HSCT due to the expansion of age limits (4, 5). The older adult populations are a heterogeneous group with differences in comorbidities associated with polypharmacy, physical and cognitive functions, nutritional status, social support, and psychological reserves of each individual (6–8). Therefore, it is challenging but crucial to identify suitable candidates for allo-HSCT regarding the risk of non-relapse mortality (NRM).

A multiparametric geriatric assessment (GA) offers comprehensive evaluations, including assessments of functional ability, physical health, cognition, psychological health, nutritional status, and social support (6–8). Prospective studies have shown that GA at diagnosis could predict non-fatal toxicities and survival for intensively treated older adults with AML; of all GA domains, physical, psychiatric, or cognitive impairments were associated with poor survival outcomes (8–10). The addition of GA domains of physical function and depression improved the predictability of the Wheatley index and treatment-related mortality scores, in addition to validating prognostic models in intensively treated older adults with AML (11). Furthermore, some studies have shown that physical or cognitive function before allo-HSCT predicted shorter overall survival (OS) (12–15). However, discrepancies among significant GA domains in each study and inclusion of relatively younger patients aged <60 years or advanced disease status at allo-HSCT have limited generalizability in older adults with AML. Furthermore, several courses of intensive chemotherapy (IC) before allo-HSCT could influence each GA domain (13–19), which may affect transplant outcomes. However, to the best of our knowledge, to date, no studies have explored the dynamic changes in GA domains before allo-HSCT and their effects on transplant outcomes. Here, we prospectively investigated the prognostic role of GA domains at diagnosis and allo-HSCT in older adults (aged ≥60 years) with AML who underwent allo-HSCT in remission and examined whether changes in GA domains affected transplant outcomes.

## 2 Materials and methods

### 2.1 Study design and GA measures

The cohort in this study was derived from a previous prospective study of GA that enrolled 105 intensively treated patients with AML aged ≥60 years and presented the predictive utility of the GA performed at diagnosis (11). In this study, we further evaluated the predictive values of pre-allo-HSCT GA among 51 patients who underwent allo-HSCT at complete remission (CR) status. Pre-allo-HSCT GA data were collected during the last follow-up visit before allo-HSCT in a median of 17 (range, 10–29) days. The GAs was performed by a study nurse who followed the published administration procedures and scored each assessment (11). All patients underwent GAs of baseline and pre-allo-HSCT GAs, which were divided into the following four categories: (I) Physical function (Korean-modified Barthel index, Korean-instrumental activities of daily living [K-IADL], and short physical performance battery [SPPB]); (II) Cognitive function (mini-mental state examination-the Korean version of Consortium to Establish a Registry for Alzheimer’s Disease Assessment Packet [MMSE-KC] and Korean nursing delirium screening scale [KNU-DESC]); (III) Psychological function (Korean version of the short form geriatric depressive scale [SGDS-K], patient health questionnaire-9, and National Comprehensive Cancer Network [NCCN] distress thermometer); and (IV) Nutritional status and social support (mini nutritional assessment [MNA] and Older Americans Resources and Services [OARS] social resources scale). Detailed GA measures and their cutoff values have been described in the previous study (11). The protocol of this study was based on the guidelines of the Institutional Review Board and was approved by the Ethics Committee of the Catholic Medical Center, Republic of Korea (KC16OISI0771). It was registered with the Clinical Research Information Service (#KCT0002172). Informed consent was obtained from all individual participants included in the study. All enrolled patients read and understood the provided information about the purpose, procedures, risks, benefits, and confidentiality of the geriatric assessment study and had the opportunity to ask questions. All patients participated voluntarily and were informed they could withdraw anytime from the study without giving a reason or bearing any cost. The study was conducted in accordance with the tenets of the Declaration of Helsinki.

### 2.2 Treatment strategy

All patients received induction chemotherapy, consisting of idarubicin (12 mg/m^2^/day) for 3 days and cytarabine (100 mg/m^2^/day) for 7 days. They received consolidation chemotherapy as a post-remission therapy after achieving CR, consisting of mitoxantrone (12 mg/m^2^/day) or idarubicin (12 mg/m^2^/day) for 3 days, along with an intermediate dose of cytarabine (1.0 g/m^2^ every 12 h) for 5 days, and these drugs were alternatively applied (20). The type of allo-HSCT conditioning regimens, either reduced-toxicity or reduced-intensity, and anti-thymocyte globulin (ATG) dosing strategy were determined by the attending physician. All patients received cyclosporin or tacrolimus with a short course of methotrexate (MTX) for graft-versus-host disease (GVHD) prophylaxis. Detailed transplantation protocols were described in supplemental materials and also in previously published manuscripts (21–23).

### 2.3 Measurable residual disease (MRD) assessments

We obtained bone marrow (BM) samples from all patients at diagnosis, and genetic mutations were screened using a real-time quantitative polymerase chain reaction (qRT-PCR) or a next-generation sequencing panel customized for acute leukemia (24). qRT-PCR was used for the *RUNX1-RUNX1T1, CBFB-MYH11*, or *NPM1* MRD assessment (25). In the absence of the aforementioned molecular targets, we used *Wilms tumor gene 1 (WT1)* transcripts as MRD markers with a positivity cut-off value of >250 copies (23). The qRT-PCR levels represented the relative ratios of *RUNX1-RUNX1T1, CBFB-MYH11, NPM1*, or *WT1* expression normalized to the expression of the reference gene *ABL1* (1 × 10^4^), as previously reported (25). We checked identified molecular target MRD levels after induction, consolidation, pre-allo-HSCT, and post-allo-HSCT at 1, 3, 6, 9, 12 months, and 1-year intervals up to 3 years after allo-HSCT.

### 2.4 Data analysis

The primary endpoint of this study was a comparison of OS, defined from the date of diagnosis to that of death for any reason or the last follow-up of censored patients, between baseline and pre-allo-HSCT GA measures. The secondary endpoint was a comparison of NRM, defined as death for any reason without evidence of disease relapse. We defined the cumulative incidence of relapse (CIR) as a relapse of AML in BM with blast >5%, the reappearance of blasts in peripheral blood, or the development of extramedullary infiltration at any site. The CIR and NRM events were observed to be in competition with each other. Disease-free survival (DFS) was defined as survival until endpoint events occurred, such as hematologic or extramedullary relapse or NRM in the CR state. CR was defined as a morphologic leukemia-free state with BM blast <5% and no persistent extramedullary disease. The distributions and frequencies of the GA measures according to each domain are presented using descriptive statistics. The chi-square test or Fisher’s exact test was performed to compare categorical variables, and the two-sample *t* test or the Wilcoxon rank sum test was performed to compare continuous variables. The directions of change between diagnostic and pre-allo-HSCT GA measures were also analyzed using the Wilcoxon rank sum test of each matched and dependent variable. The test statistic for the Wilcoxon test is W, defined as the smaller of W+ (sum of positive ranks) and W- (sum of negative ranks). In the case of multiple tests, we corrected the p-value using Bonferroni’s method. OS and DFS were estimated using the Kaplan–Meier method, and the difference in survival between the groups was compared using a log-rank analysis. We calculated NRM and CIR using a cumulative incidence estimation method, and performed between-group comparisons using Gray’s competing risk method. A multivariate analysis was performed to investigate whether the persistently impaired physical function domain was a prognostic factor, adjusted for age and donor type (variable with *P*<.100) using a Cox proportional hazards regression model for the OS. Fine–Gray proportional hazards regression was performed for the NRM. A p-value of <0.05 (two-tailed) indicated statistical significance. All statistical analyses were performed using the R software (version 3.4.1; R Foundation for Statistical Computing, Vienna, Austria).

## 3 Results

### 3.1 Baseline and allo-HSCT characteristics

The median age of the enrolled patients was 63 (range, 60–74) years, with a predominance of men (n=33, 64.7%). Nine (17.6%), 30 (58.8%), and 12 (23.5%) patients were classified into favorable, intermediate, and adverse risk categories, respectively, according to the European Leukemia Network 2022 risk classification; nine (17.6%) patients had secondary AML. All patients achieved CR, and seven (13.7%) patients had MRD-positive CR, including four (7.8%) with pre-allo-HSCT *WT1* qRT-PCR >250 copies. The median time from AML diagnosis to allo-HSCT was 6.8 (range, 3.9–8.9) months. Eleven patients (21.6%) showed a hematopoietic cell transplantation-specific comorbidity index (HCI-CI) ≥3 at diagnosis, and five more patients (n=16, 31.4%) presented with HCT-CI ≥3 before allo-HSCT. The conditioning regimen comprised reduced-toxicity (n=6, 11.8%) and reduced-intensity (n=45, 88.2%) regimens. The donor types were composed of MSDs (n=10, 19.6%), MUDs (n=14, 27.5%), and haploidentical donors (n=27, 52.9%). Cyclosporin and tacrolimus were administered to all recipients of allo-HSCT from MSD and others, respectively, with a short course of MTX as prophylaxis for GVHD. ATG doses of 5.0 mg/kg and 2.5 mg/kg were administered to 37 (72.6%) patients who underwent allo-HSCT from MUDs and haploidentical donors and 14 (27.4%) patients who underwent allo-HSCT from MSDs, respectively. **Table 1** summarizes the clinical characteristics and demographic information of the patients who underwent allo-HSCT.

**Table 1:** Clinical characteristics of the study cohort. .

Characteristics	No.
Baseline characteristics	
**Age of recipient at diagnosis (range), years**	63 years (range, 60–74)
60–64/65–70/71–75	30 (58.8%)/14 (27.5%)/7 (13.7%)
**Sex of recipient (male/female)**	33 (64.7%)/18 (35.3%)
Disease type	
*de novo* AML/secondary AML	42 (82.4%)/9 (17.6%)
ELN 2022 criteria	
Favorable/intermediate/poor	9 (17.6%)/30 (58.8%)/12 (23.5%)
Laboratory findings at baseline	
WBC, × 10^9^/L	2.68 (range, 0.49–345.72)
Hemoglobin, g/dL	9.0 (range, 6.7–12.4)
Platelet count, × 10^9^/L	62 (range, 9–268)
Basic assessment	
Cardiac function, LVEF (%)	64.0 (range, 58.4–73.0)
Pulmonary function,	
FEV1 (%)/DLCO/Adj. (%)	90.0 (range, 69.0–112.0)/78.0 (range, 42.0–119.0)
ECOG performance status 0/1	7 (13.7%)/44 (86.3%)
HCI-CI ≥3	11 (21.6%)
Allo-HSCT characteristics	
**Age of donor at allo-HSCT (median), years**	37 years (range, 20–60)
**Sex of donor (male/female)**	38 (74.5%)/13 (25.5%)
Sex mismatched/female to male	25 (49.0%)/10 (19.6%)
ABO type	
Matched	25 (49.0%)
Major/Minor/Major and minor mismatches	12 (23.5%)/10 (19.6%)/4 (7.8%)
**Time from diagnosis to allo-HSCT (median)**	6.8 months (range, 3.9–8.9)
**Number of treatment cycles prior to allo-HSCT** **Two cycles/three cycles**	12 (23.5%)/39 (76.5%)
**Pre-allo-HSCT HCT-CI ≥3**	16 (31.4%)
Pre-allo-HSCT MRD ^†^	
CR with MRD+/CR with MRD-	7 (13.7%)/44 (86.3%)
CMV IgG seropositivity	
Donor+ Recipient+/Donor- Recipient+	46 (90.2%)/5 (9.8%)
ATG dose	
2.5 mg/kg/5.0 mg/kg	14 (27.4%)/37 (72.6%)
Donor type	
MSD/MUD/Haploidentical	10 (19.6%)/14 (27.5%)/27 (52.9%)
Conditioning regimen type	
RIC/RTC	45 (88.2%)/6 (11.8%)
GVHD prophylaxis	
CS + MTX/FK + MTX	10 (19.6%)/41 (80.4%)
**CD34 infused (median)**	6.11 × 10^6^/kg (range, 1.21–17.80)

Allo-HSCT, allogeneic hematopoietic stem cell transplantation; AML, acute myeloid leukemia; ATG, anti-thymocyte globulin; CMV, cytomegalovirus; CR, complete remission; CS, cyclosporin; DLCO, diffusing capacity for carbon monoxide; ECOG, Eastern Cooperative Oncology Group; ED, early death within 60 days after induction; ELN, European leukemia network; FEV1, forced expiratory volume in one second; FK, FK506; GVHD, graft-versus-host disease; HCT-CI, hematopoietic cell transplantation-specific comorbidity index; IC, intensive chemotherapy; IQR, interquartile range; LVEF, left ventricular ejection fraction; MAC, myeloablative conditioning; MRD, minimal residual disease; MSD, matched sibling donor; MTX, methotrexate; MUD, matched unrelated donor; RIC, reduced-intensity conditioning; and RTC, reduced-toxicity conditioning.

^†^ There were seven patients with CR with MRD+ at pre-allo-HSCT workup, two patients with both NPM1 and FLT3-ITD positive, one with NPM1 positive, and four with WT1 positive of 813, 401, 332, and 312 copies (>250 copies), respectively, at pre-allo-HSCT.

### 3.2 Changes between baseline and pre-allo-HSCT GA measures

**Figure 1:**
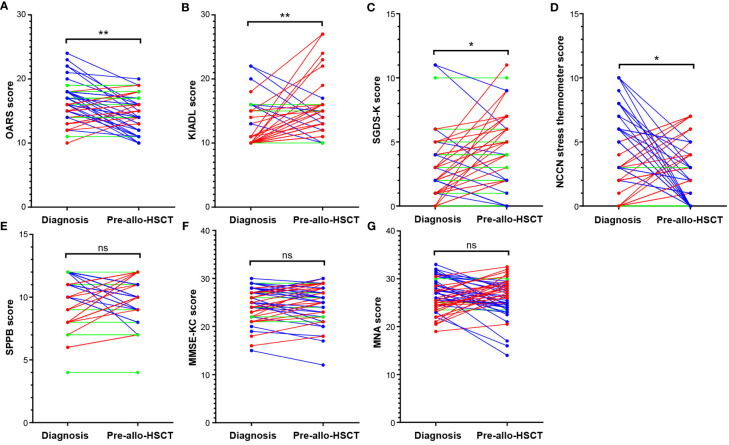
Comparison of GA measures between two different time points. Comparison of baseline and pre-allo-HSCT GA measures showed that the mean values of **(A)** OARS, **(B)** K-IADL, and **(C)** SGDS-K before allo-HSCT significantly worsened compared with those at diagnosis. However, many patients showed relief from stress before allo-HSCT, as reflected by the improved **(D)** NCCN distress thermometer readings. There were no mean differences in **(E)** physical functions (SPPB), **(F)** cognitive functions (MMSE-KC), or **(G)** nutritional status (MNA). *p<0.01, **p<0.001. Allo-HSCT, allogeneic hematopoietic stem cell transplantation; GA, geriatric assessment; K-IADL, Korean instrumental activities of daily living; MMSE-KC, mini-mental state examination-the Korean version of CERAD Assessment Packet; MNA, mini nutritional assessment; NCCN, National Comprehensive Cancer Network; OARS, Older Americans Resources and Services; SGDS-K, the Korean version of short form geriatric depressive scale. ns, not statistically significant.

The difference between the GA measures at baseline and pre-allo-HSCT of the study cohort that underwent allo-HSCT is presented in **Table 2**. **Figure 1** summarizes the difference in each GA measure between baseline and pre-allo-HSCT according to the category.

**Table 2:** Baseline and pre-allo-HSCT geriatric assessment (GA) measures.

GA score	Baseline GA N (%)		Pre-allo-HSCT GA N (%)	*p*-value
Physical function assessment				
**K-MBI as ADL measurement**				
Impaired K-MBI (≤100)	3 (5.9%)		7 (13.7%)	0.183
K-IADL				
Impaired K-IADL (≥12)	13 (25.5%)		31 (60.8%)	0.001
SPPB				
Impaired SPPB (≤8)	16 (31.4%)		10 (19.6%)	0.173
Side by side stand <10 s	1 (2.0%)		0 (0%)	1.000
Semi-tandem stand <10 s	1 (2.0%)		0 (0%)	1.000
Tandem stand <10 s	9 (17.6%)		9 (17.6%)	1.000
3.0–9.9 s	5		5	
>3.0 s or cannot perform	4		4	
Gait speed assessment, 4 meters (≥4.82 s)	26 (51.0%)		23 (45.1%)	0.552
<4.82 s (4)	25 (49.0%)		28 (54.9%)	
4.82–6.20 s (3)	14 (27.5%)		13 (25.5%)	
6.21–8.70 s (2)	8 (15.7%)		9 (17.6%)	
>8.70 s (1) or cannot perform (0)	4 (7.8%)		1 (2.0%)	
Sit-and-stand, five times (≥11.19 s)	24 (47.1%)		30 (58.8%)	0.234
<11.19 s (4)	27 (52.9%)		21 (41.2%)	
11.19–13.69 s (3)	7 (13.7%)		15 (29.4%)	
13.70–16.69 s (2)	10 (19.6%)		6 (11.8%)	
>16.7 s (1)	5 (9.8%)		8 (15.7%)	
>60 s or cannot perform (0)	2 (3.9%)		1 (2.0%)	
Nutritional status & social support assessment				
**MNA**				
Impaired MNA (≤23.5)	10 (19.6%)		5 (9.8%)	0.162
OARS				
Impaired OARS (≥18)	18 (35.3%)		27 (52.9%)	0.073
Cognition function assessment				
**MMSE-KC**				
Impaired MMSE-KC (≤23)	15 (29.4%)		16 (31.4%)	0.830
No cognitive impairment (24–30)	36 (70.6%)		35 (68.6%)	
Mild cognitive impairment (18–23)	13 (25.5%)		14 (27.5%)	
Severe cognitive impairment (0–17)	2 (3.9%)		2 (3.9%)	
KNU-DESC				
Impaired KNU-DESC (≥2)	1 (2.0%)		1 (2.0%)	1.000
Psychological function assessment				
**SGDS-K**				
Impaired SGDS-K (≥6, moderate depression)	6 (11.8%)		16 (31.4%)	0.016
No depression (0–5)	45 (88.2%)		35 (68.6%)	
Moderate depressive symptom (6–9)	3 (5.9%)		14 (27.5%)	
Major depression (≥10)	3 (5.9%)		2 (3.9%)	
NCCN distress thermometer				
Impaired NCCN distress thermometer reading (≥3)	31 (60.8%)		23 (45.1%)	0.048

ADL, Activities of daily living; K-IADL, Korean instrumental activities of daily living; K-MBI, Korean version of modified Barthel index; KNU-DESC, Korean nursing delirium screening scale; MMSE-KC, mini-mental state examination-the Korean version of CERAD assessment packet; MNA, mini Nutritional Assessment; NCCN, National Comprehensive Cancer Network; OARS, Older Americans Resources and Services; SGDS-K, the Korean version of short form geriatric depressive scale; and SPPB, short physical performance battery.

According to the Wilcoxon rank sum test, a comparison of the median of the GA measures at baseline and pre-allo-HSCT show that OARS (W -432.0 [+194.0, -626.0], p=0.003), K-IADL (W +299.0 [+430.0, -131.0], p=0.006), and SGDS-K (W +332.0 [+556.0, -224.0], p=0.018) were significantly worsened before allo-HSCT compared with those at diagnosis. Several patients demonstrated stress relief before allo-HSCT, as reflected in the improved NCCN distress thermometer readings (W -354.0 [+340.5, -694.5], p=0.044). There was no difference in physical functions (SPPB; W -22.0 [+340.5, -362.5], p=0.869), cognitive functions (MMSE-KC; W +139.0 [+587.0, -448.0], p=0.434), or nutritional status (MNA, W -95.0 [+540.5, -635.5], p=0.631). In particular, **Figure 1** shows that each patient exhibited changes in different directions, which are most noticeable in domains with no significant changes between the two time points, such as SPPB, MMSE-KC, and MNA.

### 3.3 Survival outcomes

In a median follow-up of 36.7 months (range 24.5–59.6) from allo-HSCT, the 3-year estimated OS and NRM were 60.8% (95% confidence interval [CI], 46.1%–72.6%) and 29.4% (95% CI, 17.6%–42.3%), respectively. In addition, DFS and CIR were 54.9% (95% CI, 40.6%–67.3%) and 15.7% (95% CI, 7.2%–27.1%), respectively. The cumulative incidence of grade 3–4 acute GVHD, severe chronic GVHD, and CMV disease was 13.7% (95% CI, 6.0–24.7%), 25.5% (95% CI, 14.4–38.1%), and 43.1% (95% CI, 29.2–56.4%), respectively. Twenty patients had various infections after allo-HSCT, such as bacterial septic shock (n=10), pneumonia (n=10; three fungal, three viral, and two *Pneumocystis jirovecii* infections and two atypical pneumonia with pathogen not found), herpes zoster (n=5), viral hemorrhagic cystitis (n=3), or candidemia (n=2). One patient died of veno-occlusive disease/sinusoidal obstruction syndrome. **Supplementary Table 1** summarizes allo-HSCT-related complications and survival outcomes.

### 3.4 Factors affecting survival outcomes

**Figure 2:**
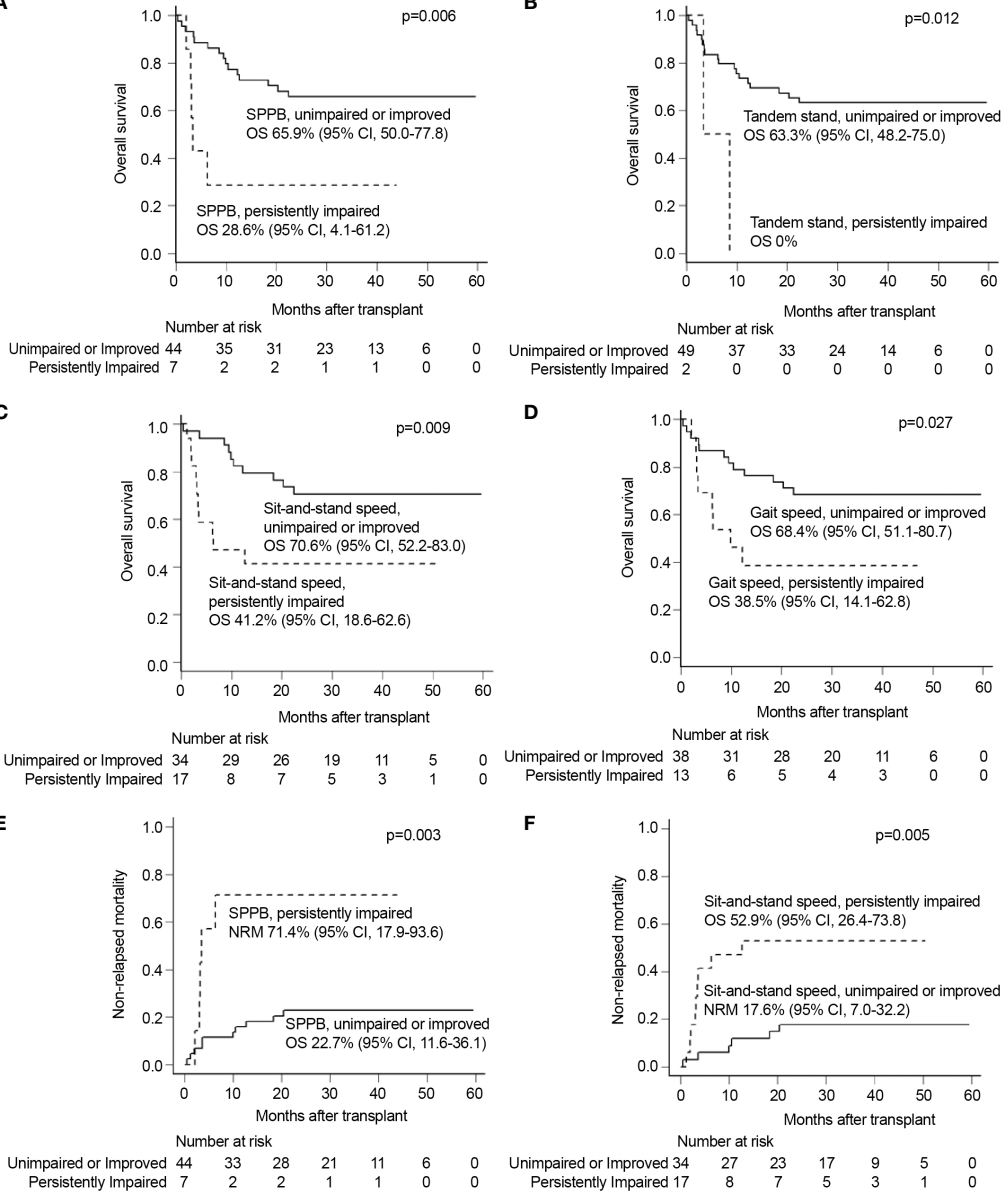
Univariable analyses of each GA measure of persistently impaired objective physical function domains with other significant covariates. Univariate analysis showed that persistent impairments of **(A)** SPPB, **(B)** tandem stand speed, **(C)** sit-and-stand speed, and **(D)** gait speed were all significantly associated with inferior OS. Persistent impairments of **(E)** SPPB and **(F)** sit-and-stand speed were also significantly related to inferior NRM. GA, geriatric assessment; NRM, non-relapse mortality; OS, overall survival; SPPB, short physical performance battery.

**Figure 3:**
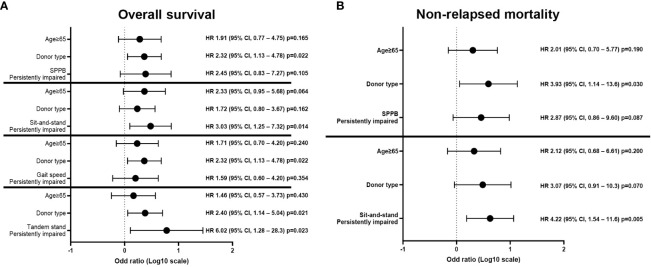
Multivariate analyses of each GA measure of persistently impaired objective physical function domains with other significant covariates. Multivariate analysis showed that persistent impairment of sit-and-stand speed was significantly associated with inferior **(A)** OS and **(B)** NRM. Persistently impaired tandem stand maneuver was also associated with poor **(A)** OS. GA, geriatric assessment; NRM, non-relapse mortality; OS, overall survival; SPPB, short physical performance battery.

The univariate analysis with baseline and transplantation-related characteristics showed that the ATG dosage (2.5 mg/kg vs. 5.0 mg/kg) and donor type (MSDs, MUDs, or haploidentical donors), where ATG dosage was determined according to donor type, were significantly associated with inferior OS (p=0.016) and higher NRM (p=0.007), respectively (**Supplementary Table 2**). Among GA measures at diagnosis (**Supplementary Figure 1**), impairment of physical function (SPPB, n=16; sit-and-stand speed, n=24; gait speed, n=26) was significantly associated with inferior OS (SPPB 43.8% vs. 68.6%, p=0.023; sit-and-stand speed 41.7% vs. 77.8%, p=0.004; and gait speed 46.2% vs. 76.0%, p=0.013) and higher NRM (sit-and-stand speed 50.0% vs. 11.1%, p=0.002; gait speed 42.3% vs. 16.0%, p=0.029) (**Supplementary Table 3**). There was no association between pre-allo-HSCT GA impairment and survival outcomes, especially SPPB and its component (**Supplementary Table 4** and **Supplementary Figure 2**). However, an analysis of changes in individual patients between diagnosis and pre-allo-HSCT revealed that persistent impairment in objectively measured physical function strongly predicted shorter OS and NRM (**Figure 2** and **Supplementary Table 5**). The baseline characteristics were not deviated according to the presence of persistent impairment between diagnosis and pre-allo-HSCT, with the exception of donor type. The donor type deviated among the persistently impaired SPPB, sit-and-stand, and gait speed groups, who mostly underwent haploidentical transplantation (**Supplementary Table 6**). Persistent impairment of physical function at pre-allo-HSCT (SPPB and tandem stand, sit-and-stand, and gait speed) was significantly associated with inferior OS (28.6% vs. 65.9%, p=0.006; 0% vs. 63.3%, p=0.012; 41.2% vs. 70.6%, p=0.009; and 38.5% vs. 68.4%, p=0.027, respectively). Persistently impaired SPPB and sit-and-stand speed were also significantly associated with higher NRM (71.4% vs. 22.7%, p=0.003; 52.9% vs. 17.6%, p=0.005). Indeed, nine patients who had impaired SPPB at diagnosis but improved SPPB at pre-allo-HSCT had better OS (55.6% vs. 28.6%, p=0.268) and reduced NRM (22.2% vs. 71.4%, p=0.117) compared with seven patients with persistently impaired SPPB at pre-allo-HSCT, without statistical significance (**Supplementary Figure 3**). Among physical function domains, patients with persistently impaired sit-and-stand maneuvers showed significantly inferior survival outcomes for both OS (hazard ratio [HR], 3.03; 95% CI, 1.25–7.32, p=0.014) and NRM (HR, 4.22; 95% CI, 1.54–11.6, p=0.005) in multivariate analysis (**Figures 3A, B**). Persistently impaired tandem stand maneuver was also associated with poor OS (HR, 6.02; 95% CI, 1.25-28.03, p=0.023, **Figure 3A**).

## 4 Discussion

This study showed that several courses of IC before allo-HSCT caused various dynamic changes in GA domains in older adults with AML, in which the directions of improvement or deterioration differed in each patient. Among all GA domains at each time point, an impairment of objectively measured physical function at diagnosis, but not at allo-HSCT, was significantly associated with shorter survival. Persistent impairment in physical function at both diagnosis and pre-allo-HSCT further strongly predicted inferior survival outcomes. Patients who experienced an improvement in physical function before allo-HSCT had similar outcomes to patients with favorable physical function at diagnosis. Other domains of GA, including patient-reported physical activity (K-IADL), were not predictive of post-transplant survival outcomes, despite dynamic changes during IC.

Physical function assessed at pre-allo-HSCT was reported to predict nonfatal to fatal treatment-related toxicities and survival outcomes after allo-HSCT (12–16, 18, 19). Three prospective studies, including various disease types, treatments, and statuses before allo-HSCT, commonly demonstrated a predictive role of objectively measured physical function, such as gait speed before allo-HSCT, for OS, despite controversies regarding the IADL indicating patient-reported physical activities (13–15). Two included younger patients, aged <60 years (13, 15). A large-scale retrospective study with older adults suggested the importance of renal dysfunction for OS but not in objectively measured physical function (12). The current prospective cohort study included only older adults aged >60 years with AML who achieved CR following IC. It should be noted that unlike earlier studies, this study evaluated dynamic changes in GA domains between diagnosis and pre-allo-HSCT. According to our previous report (11), objectively measured physical function at diagnosis was significantly associated with survival outcomes of this transplant cohort. Unlike the results of previous studies, physical function before allo-HSCT did not predict OS (13–16, 18, 19). Instead, patients with persistently impaired physical function at both diagnosis and allo-HSCT had significantly lower OS and higher NRM, while those whose physical functions improved objectively at allo-HSCT had comparable survival outcomes with those of patients with favorable physical function at both time points. Multivariate analysis showed that the prognostic value of dynamic changes in physical function during IC remained significant regardless of patient age or donor type. In particular, patients with persistently impaired sit-and-stand maneuvers before allo-HSCT had inferior outcomes in both OS and NRM, which are the most significant physical function-related variables in this cohort. Although it needs to be confirmed by further large-scale prospective analyses, these findings highlight the predictive value of dynamic changes in physical function after IC in older adults with AML who underwent allo-HSCT. Given the high rates of NRM and poor OS after allo-HSCT, patients with persistent impairment in physical function before allo-HSCT are not good candidates for allo-HSCT, but would be good candidates for maintenance therapy with oral azacitidine (26). Or, more optimized conditioning regimens may reduce NRM and improve OS in this high-risk group with sustained poor physical function. Malagola et al. published real-life data of older adults with AML and MDS in Italy from 2006 to 2017 who underwent allo-HSCT using the Busulfan or Thiotepa and Treosulfan (TREO)-based conditioning regimen. They showed that a TREO-based conditioning regimen might be preferred in patients with frail, high-risk diseases and transplants from alternative donors (27). Further studies to optimize conditioning regimens for these high-risk patients for NRM are warranted.

Favorable survival outcomes in patients who experienced improved physical function provide strong evidence for the need for interventions to maintain or improve physical function during IC in older populations. This requires evaluating interventions to improve physical function before and after allo-HSCT, such as avoiding sedative medications or polypharmacy, receiving consistent physical therapy, and utilizing assistive devices by patients with impaired physical function (28–30). Several studies on solid malignancies demonstrated that physiotherapy and nutritional counseling could improve survival, physical functioning, and quality of life (31–34). Moreover, a prehabilitation program, which includes improving a patient’s general condition before commencing treatment, was applied to patients with solid malignancies and generated promising results (35–37). The current study also revealed that, among the GA domains, SGDS-K (depression) and OARS (social support) significantly deteriorated after IC. Although these changes were not associated with OS, which could be related to a limited number of patients, they are theoretically correctable. Regarding nutritional support for intensively treated older adults with AML, Morello et al. reported that early prophylactic oral nutritional support reduced severe malnutrition incidence in post-allo-HSCT patients without side effects (38), which is worth verifying the effectiveness during induction, consolidation, and transplantation processes in elderly AML patients. Given the lack of research on AML and hematologic malignancies, well-designed prospective trials focusing on the effect of GA-directed non-oncological interventions targeting functional, nutritional, emotional, or social health will yield encouraging results and help to preserve capacity, resilience, and quality of life in older adults with AML undergoing allo-HSCT.

Cognitive impairment is another crucial aspect of GA measures that affect survival outcomes after allo-HSCT. Rebecca et al. performed a retrospective multi-institutional analysis of GA before allo-HSCT and demonstrated an association between pre-allo-HSCT cognitive deficiencies measured using the Blessed Orientation Memory Concentration test and inferior OS (16). Furthermore, a prospective study by Deschler et al. revealed that cognitive impairment after allo-HSCT was associated with decreased OS (14). Cognitive impairment is common in older adults and mild cognitive dysfunction is prevalent in those with AML, with an estimated range of 17%–35% (10). In our study, we observed cognitive impairment at diagnosis, measured using MMSE-KC, in 15 (29.4%) patients. However, 13 of 15 patients had mild cognitive dysfunction (86.7%), which did not change significantly at pre-allo-HSCT. Furthermore, there were no differences in survival outcomes according to MMSE-KC impairments at diagnosis and pre-allo-HSCT, or their dynamic changes. Our data suggest that the prognostic significance of cognitive function needs to be further validated according to the severity of the impairment and dynamic changes in older adults with AML. Moreover, the role of the caregiver should also be highlighted as an explorative topic in GA. Employed professional caregivers in hospitals or nursing homes may act appropriately against patients’ cognitive disorientation or bursting stress, which could contribute to the quality of life in elderly AML patients during treatment. Similarly, Muffly et al. mentioned that poor mental health in pre-allo-HSCT GA was associated with lower OS (15). Previously, we presented the value of depression screening in older adults with AML, represented by the baseline SGDS-K. This impairment significantly worsened survival outcomes, and adding the baseline SGDS-K improved the power of the existing survival prediction models (11). Klepin et al. reported that burdens of depressive symptoms at remission were associated with functional decline after induction chemotherapy and mortality (6). However, there is limited information on the survival impact of pre-allo-HSCT mental health deficiency. Our data showed significant overall deterioration of depressive mood at pre-allo-HSCT, but no differences in survival outcomes according to SGDS-K impairments at diagnosis and pre-allo-HSCT or their dynamic changes. Further studies are required to determine the prognostic value of depression in the setting of older adults with AML who underwent allo-HSCT.

The current study has some limitations. Given that our cohort consisted of homogeneous disease types and treatments before allo-HSCT, the modest size of the cohort may make it insufficient to detect associations between GA measures in addition to physical function and survival outcomes. Moreover, the cohort of a single institution may limit the generalizability of the results due to the inability to represent the elderly AML population undergoing allo-HSCT. Nevertheless, given the lack of studies for dynamic changes in GA domains after IC before allo-HSCT in older adults with AML, our data demonstrated the importance of repeated assessments of physical functions using objective methods to predict survival outcomes. Its prospective nature strengthens our study, and the lack of GA research on GA in Asian populations. Large-scale multicenter prospective studies are warranted to draw conclusions. In addition, further studies must address the association between impairments in the GA domains and other non-mortality outcomes crucial for older adults, including overall well-being, self-care, mobility, family support, and socialization.

In summary, we prospectively demonstrated that IC caused dynamic changes in GA domains in different directions in each older adult with AML. Our data highlight the prognostic value of physical function at diagnosis and its dynamic changes after IC, which would improve the risk stratification of the older population for transplant decisions. Given favorable survival results in patients with improved physical function during chemotherapy, more studies are needed to determine the adjustment of care for older adults with AML to improve physical function for allo-HSCT.

## Data availability statement

The datasets presented in this article are not readily available because of the nature of this research. Participants of this study disagreed about their data being shared publicly; thus, supporting data are unavailable. Requests to access the datasets should be directed to beichest@nate.com.

## Ethics statement

The protocol of this study was based on the guidelines of the Institutional Review Board and was approved by the Ethics Committee of the Catholic Medical Center, Republic of Korea (KC16OISI0771). It was registered with the Clinical Research Information Service (#KCT0002172). The studies were conducted in accordance with the local legislation and institutional requirements. The participants provided their written informed consent to participate in this study.

## Author contributions

G-JM: Data curation, Formal Analysis, Methodology, Visualization, Writing – original draft. B-SC: Conceptualization, Data curation, Formal Analysis, Funding acquisition, Supervision, Validation, Writing – review & editing. DK: Conceptualization, Data curation, Resources, Software, Validation, Visualization, Writing – review & editing. S-SP: Conceptualization, Data curation, Supervision, Validation, Writing – review & editing. SP: Data curation, Resources, Supervision, Writing – review & editing. J-HY: Software, Validation, Writing – review & editing. S-EL: Validation, Writing – review & editing. K-SE: Validation, Writing – review & editing. Y-JK: Validation, Writing – review & editing. SL: Supervision, Writing – review & editing. C-KM: Supervision, Writing – review & editing. S-GC: Supervision, Writing – review & editing. JL: Writing – review & editing. H-JK: Writing – review & editing.
