# Supplementary material for: Dynamic changes in physical function during intensive chemotherapy affect transplant outcomes in older adults with AML
Source: Front Oncol. 2023 Nov 7;13:1281782. doi: 10.3389/fonc.2023.1281782 (PMC10661959; doi:10.3389/fonc.2023.1281782)
Supplement: Supplementary file 1 [file DataSheet_1.docx]

Supplementary Material

Dynamic changes in physical function during intensive chemotherapy affect transplant outcomes in older adults with acute myeloid leukemia

Gi-June Min^1,2^, Byung-Sik Cho^1,2*^, Daehun Kwag^1,2^, Sung-Soo Park^1,2^, Silvia Park^1,2^, Jae-Ho Yoon^1,2^, Sung-Eun Lee^1,2^, Ki-Seong Eom^1,2^, Yoo-Jin Kim^1,2^, Seok Lee^1,2^, Chang-Ki Min^1,2^, Seok-Goo Cho^1^, Jong Wook Lee^1^, Hee-Je Kim^1,2^

* Correspondence: Byung-Sik Cho, MD, PhD: [cbscho@catholic.ac.kr](mailto:cbscho@catholic.ac.kr)

**1 Allogeneic hematopoietic stem cell transplantation protocol in Catholic Hematology Hospital for AML**

Patients who decided to undergo allo-HSCT received either a reduced-toxicity or reduced-intensity conditioning regimen determined by the treating physician (21). The reduced-toxicity and reduced-intensity regimen consisted of fludarabine (30 mg/m2/day) for 5 days with busulfex (3.2 mg/kg/day) for 3 days and fludarabine (30 mg/m2/day) for 5 days and busulfex (3.2 mg/kg/day) for 2 days with 400 cGy of fractionated total body irradiation (TBI), respectively. We administered anti-thymocyte globulin (ATG) at doses ranging from 1.25 to 2.5 mg/kg/day for 2 consecutive days for graft-versus-host disease (GVHD) prophylaxis in the case of human leukocyte antigen (HLA)-matched sibling donors (MSDs) and HLA-matched unrelated donors (MUDs), respectively (22). For haploidentical transplantation, we administered fludarabine (30 mg/m²/day) for 5 days and busulfan (3.2 mg/kg/day) for 2 days of fractionated TBI (total, 400 cGy) and ATG (1.25 mg/kg/day) for 4 consecutive days. All patients received GVHD prophylaxis with calcineurin inhibitors (cyclosporin in MSD and tacrolimus in MUD and haploidentical-family donors) and a short course of methotrexate on days 1, 3, 6, and 11. Calcineurin inhibitors were tapered at least 2 months after allo-HSCT with an absence of clinical GVHD and discontinued within 24 weeks after allo-HSCT. We used acyclovir and itraconazole for viral and fungal infection prophylaxes during the transplantation process. After engraftment, we maintained a six-month course of cotrimoxazole for Pneumocystis jirovecii pneumonia prophylaxis and a one-year course of acyclovir for herpes. Moreover, imaging, biopsy, and non-culture-based tests for diagnosis and prompt management of invasive fungal infections are performed with a scoring system for quality control, which is developed with important recommendations of current guidelines (23).

21. Tavaf MJ, Verkiani ME, Hanzaii FP, Zomorrod MS. Effects of immune system cells in GvHD and corresponding therapeutic strategies. Blood Res. 2023;58(1):2-12. doi: 10.5045/br.2023.2022192.

22. Cho BS, Min GJ, Park SS, Yoon SY, Park S, Jeon YW, et al. Low-dose Thymoglobulin for prevention of chronic graft-versus-host disease in transplantation from an HLA-matched sibling donor. Am J Hematol (2021) 96:1441-9. doi: 10.1002/ajh.26320.

23. Ahn H, Lee R, Cho SY, Lee DG. Advances in prophylaxis and treatment of invasive fungal infections: perspectives on hematologic diseases. Blood Res. 2022;57(S1):101-11. doi: 10.5045/br.2022.2022036.

# Supplementary Figures

**Supplementary Figure S1. Survival outcomes by baseline GA measures of objective physical function.**

Among the baseline GA measures, the physical function impairment of (A) SPPB, (B) sit-and-stand speed, and (C) gait speed were significantly associated with inferior OS. Moreover, baseline GA impairment in (D) sit-and-stand speed and (E) gait speed were significantly associated with higher NRM.

GA, geriatric assessment; NRM, non-relapse mortality; OS, overall survival; SPPB, short physical performance battery

**Supplementary Figure S2. Survival outcomes by pre-allo-HSCT GA measures of objective physical function.**

Among the pre-allo-HSCT GA measures, impairment in (A) SPPB, (B) sit-and-stand, and (C) gait speed were associated with inferior OS, although not statistically significant. Impairment in (D) sit-and-stand speed and (E) gait speed also showed a trend of higher NRM.

Allo-HSCT, allogeneic hematopoietic stem cell transplantation; GA, geriatric assessment; NRM, non-relapse mortality; OS, overall survival; SPPB, short physical performance battery

**Supplementary Figure S3. Survival outcomes of objective physical function in GA measures that improved at pre-allo-HSCT workup.**

The nine patients with impaired SPPB at diagnosis and improved at pre-allo-HSCT had superior (A) OS and (B) NRM compared with those with persistent impairment at pre-allo-HSCT, although not statistically significant.

Allo-HSCT, allogeneic hematopoietic stem cell transplantation; GA, geriatric assessment; NRM, non-relapse mortality; OS, overall survival; SPPB, short physical performance battery

# Supplementary Tables

**Supplementary Table S1. Allo-HSCT complications and clinical outcomes (n=51).**

| **Characteristics** | **% (95% CI)** |
| --- | --- |
| **Acute GVHD** |  |
| Grade 1–4 | 52.9% (38.2–65.7) |
| Grade 2–4 | 49.0% (34.6–62.0) |
| Grade 3–4 | 13.7% (6.0–24.7) |
| **Chronic GVHD** |  |
| Mild to severe | 64.7% (49.6–76.3) |
| Moderate to severe | 41.2% (27.5–54.4) |
| Severe | 25.5% (14.4–38.1) |
| **CMV DNAemia** | 66.7% (51.6–78.0) |
| **CMV disease** | 43.1% (29.2–56.3) |
| **Other infectious complications** |  |
| Bacterial septic shock, N (%) | 10 (19.6%) |
| Pneumonia, N (%) | 10 (19.6%) |
| Atypical (pathogen not found) | 2 |
| Fungal | 3 |
| Viral | 3 |
| PJP | 2 |
| Herpes zoster, N (%) | 5 (9.8%) |
| BK/JC viral hemorrhagic cystitis, N (%) | 3 (5.9%) |
| Candidemia, N (%) | 2 (3.9%) |
| **VOD/SOS**, N (%) | 1 (2.0%) |
| **Survival outcomes** |  |
| Overall survival | 60.8% (46.1–72.6) |
| Disease-free survival | 54.9% (40.3–67.3) |
| Cumulative incidence of relapse | 15.7% (7.2–27.1) |
| Non-relapsed mortality | 29.4% (17.6–42.3) |

Allo-HSCT, allogeneic hematopoietic stem cell transplantation; ATG, anti-thymocyte globulin; CMV, cytomegalovirus; GVHD, graft-versus-host disease; HCT-CI, hematopoietic cell transplantation-specific comorbidity index; PJP, *Pneumocystis jirovecii* pneumonia; and VOD/SOS, veno-occlusive disease/sinusoidal obstruction syndrome

**Supplementary Table S2. Univariate analysis of survival outcomes according to baseline and allogeneic HSCT characteristics (n=51).**

| Variables | OS | *p-*value | DFS | *p-*value | CIR | *p-*value | NRM | *p-*value |
| --- | --- | --- | --- | --- | --- | --- | --- | --- |
| **Baseline characteristics** |  |  |  |  |  |  |  |  |
| **Age** |  | 0.097 |  | 0.117 |  | 0.862 |  | 0.082 |
| 60–64 years (n=30) | 70.0% (50.3–83.1) |  | 63.3% (43.6–77.8) |  | 16.7% (5.9–32.1) |  | 20.0% (7.9–36.0) |  |
| ≥65 years (n=21) | 47.6% (25.7–66.7) |  | 42.9% (21.9–62.3) |  | 14.3% (3.3–32.9) |  | 42.9% (21.2–63.0) |  |
| **ELN 2022 risk stratification** |  | 0.491 |  | 0.320 |  | 0.923 |  | 0.385 |
| Favorable risk (n=9) | 77.8% (36.5–93.9) |  | 77.8% (36.5–93.9) |  | 11.1% (0.5–40.6) |  | 11.1% (0.5–40.9) |  |
| Intermediate risk (n=30) | 56.7% (37.3–72.1) |  | 50.0% (31.3–66.1) |  | 16.7% (5.9–32.2) |  | 33.3% (17.2–50.4) |  |
| Poor risk (n=12) | 58.3% (27.0–80.1) |  | 50.0% (20.8–73.6) |  | 16.7% (2.2–43.1) |  | 33.3% (9.3–60.1) |  |
| **Sex** |  | 0.449 |  | 0.465 |  | 0.149 |  | 0.078 |
| Male (n=33) | 63.6% (44.9–77.5) |  | 57.6% (39.1–72.3) |  | 21.2% (9.2–36.6) |  | 21.2% (9.2–36.6) |  |
| Female (n=18) | 55.6% (30.5–74.8) |  | 50.0% (25.9–70.1) |  | 5.6% (0.3–23.4) |  | 44.4% (20.7–65.9) |  |
| **Disease type** |  | 0.450 |  | 0.356 |  | 0.769 |  | 0.405 |
| *de novo* AML (n=41) | 63.4% (46.8–76.1) |  | 58.5% (42.0–71.8) |  | 14.6% (5.8–27.3) |  | 26.8% (14.3–41.0) |  |
| Secondary AML* (n=10) | 50.0% (18.4–75.3) |  | 40.0% (12.3–67.0) |  | 20.0% (2.3–50.2) |  | 40.0% (10.8–68.5) |  |
| **ECOG-performance status (≥2, impaired)** |  | 0.478 |  | 0.436 |  | 0.453 |  | 0.389 |
| Unimpaired (n=50) | 60.0% (45.1–72.0) |  | 54.0% (39.3–66.6) |  | 16.0% (7.4–27.5) |  | 30.0% (17.9–43.0) |  |
| Impaired (n=1) | 100% |  | 100% |  | 0% |  | 0% |  |
| **Pre-allogeneic HSCT characteristics** |  |  |  |  |  |  |  |  |
| **HCI-CI** |  | 0.243 |  | 0.447 |  | 0.142 |  | 0.063 |
| 0–2 (n=41) | 63.4% (46.8–761) |  | 56.1% (39.7–69.6) |  | 19.5% (9.0–33.0) |  | 24.4% (12.5–38.4) |  |
| ≥3 (n=10) | 50.0% (18.4–75.3) |  | 50.0% (18.4–75.3) |  | 0% |  | 50.0% (16.3–76.8) |  |
| **Donor to recipient sex mismatch** |  | 0.224 |  | 0.164 |  | 0.952 |  | 0.113 |
| No (n=26) | 69.2% (47.8–83.3) |  | 65.4% (44.0–80.3) |  | 15.4% (4.7–31.9) |  | 19.2% (6.8–36.4) |  |
| Yes (n=25) | 52.0% (31.2–69.2) |  | 44.0% (24.5–61.9) |  | 16.0% (4.8–33.2) |  | 40.0% (20.8–58.6) |  |
| **ABO matching degree** |  | 0.540 |  | 0.509 |  | 0.199 |  | 0.777 |
| Match or minor mismatch (n=35) | 57.1% (39.3–71.5) |  | 51.4% (34.0–66.4) |  | 20.0% (8.6–34.7) |  | 28.6% (14.7–44.1) |  |
| Major or major/minor mismatch (n=16) | 68.8% (40.5–85.6) |  | 62.5% (34.9–81.1) |  | 6.2% (0.3–25.9) |  | 31.2% (10.8–54.5) |  |
| **Conditioning intensity** |  | 0.648 |  | 0.937 |  | 0.018 |  | 0.104 |
| RTC (n=6) | 66.7% (19.5–90.4) |  | 50.0% (11.1–80.4) |  | 50.0% (7.7–82.9) |  | 0% |  |
| RIC (n=45) | 60.0% (44.3–72.6) |  | 55.6% (40.0–68.6) |  | 11.1% (4.0–22.3) |  | 33.3% (20.0–47.2) |  |
| **ATG dosage** |  | 0.016 |  | 0.082 |  | 0.806 |  | 0.086 |
| 2.5 mg/kg (n=14) | 86.7% (56.4–96.5) |  | 73.3% (43.6–89.1) |  | 13.3% (2.0–35.4) |  | 13.3% (2.0–35.6) |  |
| 5.0 mg/kg (n=37) | 50.0% (32.9–64.9) |  | 47.2% (30.5–62.3) |  | 16.7% (6.6–30.7) |  | 36.1% (20.7–51.7) |  |
| **Donor type** |  | 0.053 |  | 0.205 |  | 0.005 |  | 0.007 |
| MSD (n=10) | 90.0% (47.3–98.5) |  | 80.0% (40.9–94.6) |  | 10.0% (0.5–37.4) |  | 10.0% (0.4–37.6) |  |
| MUD (n=14) | 64.3% (34.3–83.3) |  | 50.0% (22.9–72.2) |  | 42.9% (16.6–67.1) |  | 7.1% (0.4–28.9) |  |
| Haploidentical (n=27) | 48.1% (28.7–65.2) |  | 48.1% (28.7–65.2) |  | 3.7% (0.2–16.5) |  | 48.1% (28.2–65.6) |  |

AML, acute myeloid leukemia; ATG, anti-thymocyte globulin; CIR, cumulative incidence of relapse; DFS, disease-free survival; ECOG, Eastern Cooperative Oncology Group; ELN, European Leukemia Network; HCT-CI, hematopoietic cell transplantation-specific comorbidity index; MAC, myeloablative conditioning; MSD, matched sibling donor; MUD, matched unrelated donor; OS, overall survival; RIC, reduced-intensity conditioning; RTC, reduced-toxicity conditioning; NRM, non-relapse mortality

**Supplementary Table S3. Univariate analysis of survival outcomes according to baseline GA measures at diagnosis in patients who underwent allo-HSCT (n=51).**

| Variables | OS | *p-*value | DFS | *p-*value | CIR | *p-*value | NRM | *p-*value |
| --- | --- | --- | --- | --- | --- | --- | --- | --- |
| **Physical function assessment** |  |  |  |  |  |  |  |  |
| **K-MBI (≤100, impaired)** |  | 0.324 |  | 0.441 |  | 0.471 |  | 0.185 |
| Unimpaired (n=48) | 62.5% (47.3–74.5) |  | 56.2% (41.2–68.9) |  | 16.7% (7.7–28.6) |  | 27.1% (15.4–40.2) |  |
| Impaired (n=3) | 33.3% (0.9–77.4) |  | 33.3% (0.9–77.4) |  | 0% |  | 66.7% (0.2–97.3) |  |
| **K-IADL (≥12, impaired)** |  | 0.767 |  | 0.850 |  | 0.842 |  | 0.987 |
| Unimpaired (n=37) | 62.2% (44.6–75.6) |  | 54.1% (36.9–68.4) |  | 16.2% (6.4–29.9) |  | 29.7% (15.9–44.9) |  |
| Impaired (n=14) | 57.1% (28.4–78.0) |  | 57.1% (28.4–78.0) |  | 14.3% (2.0–37.9) |  | 28.6% (8.2–53.4) |  |
| **SPPB (≤8, impaired)** |  | 0.023 |  | 0.085 |  | 0.726 |  | 0.076 |
| Unimpaired (n=35) | 68.6% (50.5–81.2) |  | 60.0% (42.0–74.0) |  | 17.1% (6.8–31.4) |  | 22.9% (10.6–37.9) |  |
| Impaired (n=16) | 43.8% (19.8–65.6) |  | 43.8% (19.8–65.6) |  | 12.5% (1.8–34.1) |  | 43.8% (18.8–66.4) |  |
| **Tandem stand (<10 s)** |  | 0.250 |  | 0.434 |  | 0.610 |  | 0.312 |
| Unimpaired (n=48) | 63.4% (46.8–76.1) |  | 56.1% (39.7–69.6) |  | 17.1% (7.4–30.2) |  | 26.8% (14.3–41.0) |  |
| Impaired (n=10) | 50.0% (18.4–75.3) |  | 50.0% (18.4–75.3) |  | 10.0% (0.4–38.6) |  | 40.0% (10.8–68.5) |  |
| **Sit-and-stand, five times (≥11.20 s)** |  | 0.004 |  | 0.038 |  | 0.187 |  | 0.002 |
| Unimpaired (n=27) | 77.8% (57.1–89.3) |  | 66.7% (45.7–81.1) |  | 22.2% (8.8–39.4) |  | 11.1% (2.7–26.3) |  |
| Impaired (n=24) | 41.7% (22.2–60.1) |  | 41.7% (22.2–60.1) |  | 8.3% (1.3–24.0) |  | 50.0% (28.4–68.3) |  |
| **Gait speed (≥4.82 s)** |  | 0.013 |  | 0.092 |  | 0.436 |  | 0.029 |
| Unimpaired (n=25) | 76.0% (54.2–88.4) |  | 64.0% (42.2–79.4) |  | 20.0% (7.0–37.7) |  | 16.0% (4.8–33.0) |  |
| Impaired (n=26) | 46.2% (26.6–63.6) |  | 46.2% (26.6–63.6) |  | 11.5% (2.7–27.3) |  | 42.3% (23.0–60.5) |  |
| **Nutritional status & social support** |  |  |  |  |  |  |  |  |
| **MNA (≤23.5, malnutrition)** |  | 0.864 |  | 0.813 |  | 0.857 |  | 0.788 |
| Nourished group (n=40) | 62.5% (45.7–75.4) |  | 55.0% (38.5–68.8) |  | 15.0% (6.0–27.9) |  | 30.0% (16.6–44.6) |  |
| Malnutrition at-risk group (n=11) | 54.5% (22.9–78.0) |  | 54.5% (22.9–78.0) |  | 18.2% (2.4–46.0) |  | 27.3% (5.8–55.3) |  |
| **OARS (≥18, impaired)** |  | 0.476 |  | 0.759 |  | 0.475 |  | 0.761 |
| Unimpaired (n=33) | 57.6% (39.1–72.3) |  | 54.5% (36.3–69.6) |  | 18.2% (7.2–33.1) |  | 27.3% (13.4–43.2) |  |
| Impaired (n=18) | 66.7% (40.4–83.4) |  | 55.6% (30.5–74.8) |  | 11.1% (1.7–30.7) |  | 33.3% (13.1–55.3) |  |
| **Cognition assessment** |  |  |  |  |  |  |  |  |
| **MMSE-KC (≤23, impaired)** |  | 0.250 |  | 0.295 |  | 0.771 |  | 0.333 |
| Unimpaired (n=36) | 55.6% (38.1–69.9) |  | 50.0% (32.9–64.9) |  | 16.7% (6.6–30.7) |  | 33.3% (18.5–48.9) |  |
| Impaired (n=15) | 73.3% (43.6–89.1) |  | 66.7% (37.5–84.6) |  | 13.3% (2.0–35.6) |  | 20.0% (4.5–43.3) |  |
| **KNU-DESC (≥2, impaired)** |  | 0.478 |  | 0.438 |  | 0.453 |  | 0.389 |
| Unimpaired (n=50) | 60.0% (45.1–72.0) |  | 54.0% (39.3–66.6) |  | 16.0% (7.4–27.5) |  | 30.0% (17.9–43.0) |  |
| Impaired (n=1) | 100% |  | 100% |  | 0% |  | 0% |  |
| **Psychological function assessment** |  |  |  |  |  |  |  |  |
| **SGDS-K (≥6, impaired)** |  | 0.760 |  | 0.825 |  | 0.953 |  | 0.841 |
| Unimpaired (n=45) | 60.0% (44.3–72.6) |  | 55.6% (40.0–68.6) |  | 15.6% (6.7–27.7) |  | 28.9% (16.4–42.6) |  |
| Impaired (n=6) | 66.7% (919.5–90.4) |  | 50.0% (11.1–80.4) |  | 16.7% (0.4–55.6) |  | 33.3% (3.0–71.0) |  |
| **NCCN distress thermometer reading (≥3, impaired)** |  | 0.499 |  | 0.520 |  | 0.958 |  | 0.518 |
| Unimpaired (n=20) | 55.0% (31.3–73.5) |  | 50.0% (27.1–69.2) |  | 15.0% (3.5–34.2) |  | 35.0% (15.1–55.9) |  |
| Impaired (n=31) | 64.5% (45.2–78.5) |  | 58.1% (39.0–73.1) |  | 16.1% (5.7–31.2) |  | 25.8% (12.0–42.2) |  |

ADL, activities of daily living; CIR, cumulative incidence of relapse; DFS, disease-free survival; K-IADL, Korean instrumental activities of daily living; K-MBI, Korean version of modified Barthel index; KNU-DESC, Korean nursing delirium screening scale; MMSE-KC, mini-mental state examination-the Korean version of CERAD assessment packet; MNA, mini nutritional assessment; NCCN, National Comprehensive Cancer Network; NRM, non-relapse mortality; OARS, Older Americans Resources and Services; OS, overall survival; SGDS-K, the Korean version of short form geriatric depressive scale; SPPB, short physical performance battery

**Supplementary Table S4. Univariate analysis of survival outcomes according to pre-allo-HSCT GA measures in patients who underwent allo-HSCT (n=51).**

| Variables | OS | *p-*value | DFS | *p-*value | CIR | *p-*value | NRM | *p-*value |
| --- | --- | --- | --- | --- | --- | --- | --- | --- |
| **Physical function assessment** |  |  |  |  |  |  |  |  |
| **K-MBI (≤100, impaired)** |  | 0.244 |  | 0.061 |  | 0.264 |  | 0.405 |
| Unimpaired (n=44) | 63.6% (47.7–75.9) |  | 59.1% (43.2–71.9) |  | 13.6% (5.4–25.6) |  | 27.3% (15.1–41.0) |  |
| Impaired (n=7) | 42.9% (9.8–73.4) |  | 28.6% (4.1–61.2) |  | 28.6% (2.7–64.8) |  | 42.9% (6.7–76.6) |  |
| **K-IADL (≥12, impaired)** |  | 0.399 |  | 0.222 |  | 0.503 |  | 0.445 |
| Unimpaired (n=20) | 55.0% (31.3–73.5) |  | 45.0% (23.1–64.7) |  | 20.0% (5.8–40.2) |  | 35.0% (15.1–55.9) |  |
| Impaired (n=31) | 64.5% (45.2–78.5) |  | 61.3% (42.0–75.8) |  | 12.9% (3.9–27.3) |  | 25.8% (12.0–42.1) |  |
| **SPPB (≤8, impaired)** |  | 0.247 |  | 0.461 |  | 0.143 |  | 0.082 |
| Unimpaired (n=41) | 63.4% (46.8–76.1) |  | 56.1% (39.7–69.6) |  | 19.5% (9.0–33.0) |  | 24.4% (12.5–38.4) |  |
| Impaired (n=10) | 50.0% (18.4–75.3) |  | 50.0% (18.4–75.3) |  | 0% |  | 50.0% (16.3–76.8) |  |
| **Tandem stand (<10 s)** |  | 0.674 |  | 0.993 |  | 0.095 |  | 0.204 |
| Unimpaired (n=42) | 59.5% (43.2–72.6) |  | 54.8% (38.7–683) |  | 11.9% (4.3–23.8) |  | 33.3% (19.6–47.7) |  |
| Impaired (n=9) | 66.7% (28.2–87.8) |  | 55.6% (20.4–80.5) |  | 33.3% (6.5–64.3) |  | 11.1% (0.5–40.6) |  |
| **Sit-and-stand, five times (≥11.20 s)** |  | 0.720 |  | 0.985 |  | 0.069 |  | 0.166 |
| Unimpaired (n=21) | 61.9% (38.1–78.8) |  | 52.4% (29.7–70.9) |  | 28.6% (11.2–48.9) |  | 19.0% (5.7–38.3) |  |
| Impaired (n=30) | 60.0% (40.5–75.0) |  | 56.7% (37.3–72.1) |  | 6.7% (1.1–19.5) |  | 36.7% (19.8–53.7) |  |
| **Gait speed (≥4.82 s)** |  | 0.471 |  | 0.257 |  | 0.719 |  | 0.401 |
| Unimpaired (n=28) | 64.3% (43.8–78.9) |  | 60.7% (40.4–76.0) |  | 14.3% (4.3–29.9) |  | 25.0% (10.8–42.2) |  |
| Impaired (n=23) | 56.5% (34.4–73.8) |  | 47.8% (26.8–66.1) |  | 17.4% (5.2–35.6) |  | 34.8% (16.1–54.3) |  |
| **Nutritional status & social support** |  |  |  |  |  |  |  |  |
| **MNA (≤23.5, malnutrition)** |  | 0.987 |  | 0.794 |  | 0.323 |  | 0.647 |
| Nourished group (n=46) | 60.9% (45.3–73.3) |  | 54.3% (39.0–67.4) |  | 17.4% (8.0–29.7) |  | 28.3% (16.1–41.8) |  |
| Malnutrition at-risk group (n=5) | 60.0% (12.6–88.2) |  | 60.0% (12.6–88.2) |  | 0% |  | 40.0% (3.1–78.6) |  |
| **OARS (≥18, impaired)** |  | 0.325 |  | 0.491 |  | 0.547 |  | 0.240 |
| Unimpaired (n=24) | 54.2% (32.7–71.4) |  | 50.0% (29.1–67.8) |  | 12.5% (3.0–29.3) |  | 37.5% (18.5–56.5) |  |
| Impaired (n=27) | 66.7% (45.7–81.1) |  | 59.3% (38.6–75.0) |  | 18.5% (6.5–35.3) |  | 22.2% (8.8–39.4) |  |
| **Cognition assessment** |  |  |  |  |  |  |  |  |
| **MMSE-KC (≤23, impaired)** |  | 0.059 |  | 0.051 |  | 0.061 |  | 0.270 |
| Unimpaired (n=35) | 51.4% (34.0–66.4) |  | 42.9% (26.4–58.3) |  | 22.9% (10.5–38.0) |  | 34.3% (19.1–50.1) |  |
| Impaired (n=16) | 81.2% (52.5–93.5) |  | 81.2% (52.5–93.5) |  | 0% |  | 18.8% (4.3–41.0) |  |
| **KNU-DESC (≥2, impaired)** |  | 0.478 |  | 0.436 |  | 0.453 |  | 0.389 |
| Unimpaired (n=50) | 60.0% (45.1–72.0) |  | 54.0% (39.3–66.6) |  | 16.0% (7.4–27.5) |  | 30.0% (17.9–43.0) |  |
| Impaired (n=1) | 100% |  | 100% |  | 0% |  | 0% |  |
| **Psychological function assessment** |  |  |  |  |  |  |  |  |
| **SGDS-K (≥6, impaired)** |  | 0.483 |  | 0.999 |  | 0.623 |  | 0.642 |
| Unimpaired (n=35) | 57.1% (39.3–71.5) |  | 54.3% (36.6–69.0) |  | 14.3% (5.1–28.1) |  | 31.4% (16.9–47.1) |  |
| Impaired (n=16) | 68.8% (40.5–85.6) |  | 56.2% (29.5–76.2) |  | 18.8% (4.2–41.2) |  | 25.0% (7.3–48.1) |  |
| **NCCN distress thermometer reading (≥3, impaired)** |  | 0.653 |  | 0.671 |  | 0.064 |  | 0.333 |
| Unimpaired (n=28) | 57.1% (37.1–72.9) |  | 57.1% (37.1–72.9) |  | 7.1% (1.2–20.8) |  | 35.7% (18.5–53.4) |  |
| Impaired (n=23) | 65.2% (42.3–80.8) |  | 52.2% (30.5–70.0) |  | 26.1% (10.2–45.3) |  | 21.7% (7.6–40.4) |  |

ADL, activities of daily living; CIR, cumulative incidence of relapse; DFS, disease-free survival; K-IADL, Korean instrumental activities of daily living; K-MBI, Korean version of modified Barthel index; KNU-DESC, Korean nursing delirium screening scale; MMSE-KC, mini-mental state examination-the Korean version of CERAD assessment packet; MNA, mini nutritional assessment; NRM, non-relapse mortality; NCCN, National Comprehensive Cancer Network; OARS, Older Americans Resources and Services; OS, overall survival; SGDS-K, the Korean version of short form geriatric depressive scale; SPPB, short physical performance battery

**Supplementary Table S5. Univariate analysis of survival outcomes according to persistently impaired outcomes of GA measures before transplantation in patients who underwent allo-HSCT (n=51)**

| Variables | OS | *p-*value | DFS | *p-*value | CIR | *p-*value | NRM | *p-*value |
| --- | --- | --- | --- | --- | --- | --- | --- | --- |
| **Physical function assessment** |  |  |  |  |  |  |  |  |
| **K-MBI (≤100, impaired)** |  | 0.854 |  | 0.994 |  | 0.546 |  | 0.607 |
| Others (n=49) | 61.2% (46.2–73.2) |  | 55.1% (40.2–67.7) |  | 16.3% (7.5–28.1) |  | 28.6% (16.7–41.7) |  |
| Persistently impaired (n=2) | 50.0% (59.8–91.0) |  | 50.0% (59.8–91.0) |  | 0% |  | 50.0% (0–96.0) |  |
| **K-IADL (≥12, impaired)** |  | 0.959 |  | 0.703 |  | 0.944 |  | 0.687 |
| Others (n=39) | 61.5% (44.5–74.7) |  | 53.8% (37.2–67.9) |  | 15.4% (6.1–28.5) |  | 30.8% (17.0–45.6) |  |
| Persistently impaired (n=12) | 58.3% (27.0–80.1) |  | 58.3% (27.0–80.1) |  | 16.7% (2.3–42.9) |  | 25.0% (5.4–51.7) |  |
| **SPPB (≤8, impaired)** |  | 0.006 |  | 0.019 |  | 0.263 |  | 0.003 |
| Others (n=44) | 65.9% (50.0–77.8) |  | 59.1% (43.2–71.9) |  | 18.2% (8.4–30.9) |  | 22.7% (11.6–36.1) |  |
| Persistently impaired (n=7) | 28.6% (4.1–61.2) |  | 28.6% (4.1–61.2) |  | 0% |  | 71.4% (17.9–93.6) |  |
| **Tandem stand (<10 s)** |  | 0.012 |  | 0.012 |  | 0.012 |  | 0.255 |
| Others (n=49) | 63.3% (48.2–75.0) |  | 57.1% (42.2–69.6) |  | 14.3% (6.2–25.6) |  | 28.6% (16.7–41.7) |  |
| Persistently impaired (n=2) | 0% |  | 0% |  | 50.0% (0–98.7) |  | 50.0% (0–96.0) |  |
| **Sit-and-stand, five times (≥11.20 s)** |  | 0.009 |  | 0.046 |  | 0.198 |  | 0.005 |
| Others (n=34) | 70.6% (52.2–83.0) |  | 61.8% (43.4–75.7) |  | 20.6% (8.9–35.6) |  | 17.6% (7.0–32.2) |  |
| Persistently impaired (n=17) | 41.2% (18.6–62.6) |  | 41.2% (18.6–62.6) |  | 5.9% (0.3–24.7) |  | 52.9% (26.4–73.8) |  |
| **Gait speed (≥4.82 s)** |  | 0.027 |  | 0.092 |  | 0.987 |  | 0.108 |
| Others (n=38) | 68.4% (51.1–80.7) |  | 60.5% (43.3–74.0) |  | 15.8% (6.3–29.2) |  | 23.7% (11.6–38.2) |  |
| Persistently impaired (n=13) | 38.5% (14.1–62.8) |  | 38.5% (14.1–62.8) |  | 15.4% (2.0–40.7) |  | 46.2% (17.7–70.8) |  |
| **Nutritional status & social support** |  |  |  |  |  |  |  |  |
| **MNA (≤23.5, malnutrition)** |  | 0.332 |  | 0.451 |  | 0.467 |  | 0.176 |
| Others (n=48) | 62.5% (47.3–74.5) |  | 56.2% (41.2–68.9) |  | 16.7% (7.7–28.6) |  | 27.1% (15.4–40.2) |  |
| Persistently impaired (n=3) | 33.3% (8.9–77.4) |  | 33.3% (0.8–77.4) |  | 0% |  | 66.7% (0.2–97.3) |  |
| **OARS (≥18, impaired)** |  | 0.155 |  | 0.313 |  | 0.906 |  | 0.307 |
| Others (n=44) | 56.8% (41.0–69.9) |  | 52.3% (36.7–65.7) |  | 15.9% (6.9–28.3) |  | 31.8% (18.6–45.8) |  |
| Persistently impaired (n=7) | 85.7% (33.4–97.9) |  | 71.4% (25.8–92.0) |  | 14.3% (0.5–49.1) |  | 14.3% (0.5–49.6) |  |
| **Cognition assessment** |  |  |  |  |  |  |  |  |
| **MMSE-KC (≤23, impaired)** |  | 0.059 |  | 0.051 |  | 0.135 |  | 0.156 |
| Others (n=41) | 53.7% (37.4–67.4) |  | 46.3% (30.7–60.6) |  | 19.5% (9.0–33.0) |  | 34.1% (20.0–48.8) |  |
| Persistently impaired (n=10) | 90.0% (47.3–98.3) |  | 90.0% (47.3–98.5) |  | 0% |  | 10.0% (0.5–37.4) |  |
| **KNU-DESC (≥2, impaired)** |  | – |  | – |  | – |  | – |
| Others (n=51) | 60.8% (46.1–72.6) |  | 54.9% (40.3–67.3) |  | 15.7% (7.2–27.1) |  | 29.4% (17.6–42.3) |  |
| Persistently impaired (n=0) | – |  | – |  | – |  | – |  |
| **Psychological function assessment** |  |  |  |  |  |  |  |  |
| **SGDS-K (≥6, impaired)** |  | 0.344 |  | 0.702 |  | 0.792 |  | 0.568 |
| Others (n=46) | 58.7% (43.2–71.3) |  | 54.3% (39.0–67.4) |  | 15.2% (6.6–27.2) |  | 30.4% (17.8–44.1) |  |
| Persistently impaired (n=5) | 80.0% (20.4–96.9) |  | 60.0% (12.6–88.2) |  | 20.0% (0.4–62.1) |  | 20.0% (0.4–63.2) |  |
| **NCCN distress thermometer reading (≥3, impaired)** |  | 0.692 |  | 0.858 |  | 0.608 |  | 0.840 |
| Others (n=36) | 58.3% (40.7–72.4) |  | 55.6% (38.1–69.9) |  | 13.9% (5.0–27.3) |  | 30.6% (16.4–46.0) |  |
| Persistently impaired (n=15) | 66.7% (37.5–84.6) |  | 53.3% (26.3–74.4) |  | 20.0% (4.4–43.6) |  | 26.7% (7.7–50.6) |  |

ADL, activities of daily living; CIR, cumulative incidence of relapse; DFS, disease-free survival; K-IADL, Korean instrumental activities of daily living; K-MBI, Korean version of modified Barthel index; KNU-DESC, Korean nursing delirium screening scale; MMSE-KC, mini-mental state examination-the Korean version of CERAD assessment packet; MNA, mini nutritional assessment; NCCN, National Comprehensive Cancer Network; NRM, non-relapse mortality; OARS, Older Americans Resources and Services; OS, overall survival; SGDS-K, the Korean version of short form geriatric depressive scale; SPPB, short physical performance battery

**Supplementary Table S6. Baseline characteristics sorted by GA categories at pre-allo-HSCT**

| GA categories | Intact | Persistently impaired | *p* values |
| --- | --- | --- | --- |
| **K-MBI as ADL assessment (≤100, impaired)** | N=49 | N=2 |  |
| Age ≥65 years | 21 (42.9%) | 0 (0%) | 0.506 |
| ELN 2022 risk |  |  |  |
| Low risk | 9 (18.4%) | 0 (0%) | 1.000 |
| Intermediate risk | 29 (59.2%) | 1 (50.0%) | 1.000 |
| High risk | 11 (22.4%) | 1 (50.0%) | 0.419 |
| Female sex | 17 (34.7%) | 1 (50.0%) | 1.000 |
| Secondary AML | 9 (18.4%) | 1 (50.0%) | 0.357 |
| HCI-CI ≥3 | 9 (18.4%) | 1 (50.0%) | 0.357 |
| Sex mismatch | 24 (49.0%) | 1 (50.0%) | 1.000 |
| ABO match | 35 (71.4%) | 0 (0%) | 0.094 |
| RIC | 43 (87.8%) | 2 (100%) | 1.000 |
| ATG dosage 5.0 mg/kg | 36 (73.5%) | 1 (50.0%) | 0.478 |
| Donor type |  |  |  |
| MSD | 10 (20.4%) | 0 (0%) | 1.000 |
| MUD | 13 (26.5%) | 1 (50.0%) | 0.478 |
| Haploidentical | 26 (53.1%) | 1 (50.0%) | 1.000 |
| **K-IADL (≥12, impaired)** | N=39 | N=12 |  |
| Age ≥65 years | 16 (41.0%) | 5 (41.7%) | 1.000 |
| ELN 2022 risk |  |  |  |
| Low risk | 6 (15.4%) | 3 (25.0%) | 0.445 |
| Intermediate risk | 23 (59.0%) | 7 (58.3%) | 1.000 |
| High risk | 10 (25.6%) | 2 (16.7%) | 0.706 |
| Female sex | 16 (41.0%) | 2 (16.7%) | 0.174 |
| Secondary AML | 8 (20.5%) | 2 (16.7%) | 1.000 |
| HCI-CI ≥3 | 8 (20.5%) | 2 (16.7%) | 1.000 |
| Sex mismatch | 20 (51.3%) | 5 (41.7%) | 0.560 |
| ABO match | 27 (69.2%) | 8 (66.7%) | 1.000 |
| RIC | 35 (89.7%) | 10 (83.3%) | 0.616 |
| ATG dosage 5.0 mg/kg | 29 (74.4%) | 8 (66.7%) | 0.715 |
| Donor type |  |  |  |
| MSD | 6 (15.4%) | 4 (33.3%) | 0.218 |
| MUD | 10 (25.6%) | 4 (33.3%) | 0.715 |
| Haploidentical | 23 (59.0%) | 4 (33.3%) | 0.120 |
| **SPPB (≤8, impaired)** | N=44 | N=7 |  |
| Age ≥65 years | 17 (38.6%) | 4 (57.1%) | 0.427 |
| ELN 2022 risk |  |  |  |
| Low risk | 9 (20.5%) | 0 (0%) | 0.328 |
| Intermediate risk | 25 (56.8%) | 5 (71.4%) | 0.685 |
| High risk | 10 (22.7%) | 2 (28.6%) | 0.662 |
| Female sex | 13 (29.5%) | 5 (71.4%) | 0.082 |
| Secondary AML | 8 (18.2%) | 2 (28.6%) | 0.612 |
| HCI-CI ≥3 | 7 (15.9%) | 3 (42.9%) | 0.126 |
| Sex mismatch | 20 (45.5%) | 5 (71.4%) | 0.248 |
| ABO match | 30 (68.2%) | 5 (71.4%) | 1.000 |
| RIC | 38 (86.4%) | 7 (100%) | 0.578 |
| ATG dosage 5.0 mg/kg | 30 (68.2%) | 7 (100%) | 0.169 |
| Donor type |  |  |  |
| MSD | 10 (22.7%) | 0 (0%) | 0.320 |
| MUD | 14 (31.8%) | 0 (0%) | 0.169 |
| Haploidentical | 20 (45.5%) | 7 (100%) | 0.011 |
| **Tandem stand (<10 s)** | N=49 | N=2 |  |
| Age ≥65 years | 19 (38.8%) | 2 (100%) | 0.165 |
| ELN 2022 risk |  |  |  |
| Low risk | 9 (18.4%) | 0 (0%) | 1.000 |
| Intermediate risk | 28 (57.1%) | 2 (100%) | 0.506 |
| High risk | 12 (24.5%) | 0 (0%) | 1.000 |
| Female sex | 18 (36.7%) | 0 (0%) | 0.534 |
| Secondary AML | 10 (20.4%) | 0 (0%) | 1.000 |
| HCI-CI ≥3 | 9 (18.4%) | 1 (50.0%) | 0.357 |
| Sex mismatch | 25 (51.0%) | 0 (0%) | 0.490 |
| ABO match | 33 (67.3%) | 2 (100%) | 1.000 |
| RIC | 43 (87.8%) | 2 (100%) | 1.000 |
| ATG dosage 5.0 mg/kg | 35 (71.4%) | 2 (100%) | 1.000 |
| Donor type |  |  |  |
| MSD | 10 (20.4%) | 0 (0%) | 1.000 |
| MUD | 13 (26.5%) | 1 (50.0%) | 0.478 |
| Haploidentical | 26 (53.1%) | 1 (50.0%) | 1.000 |
| **Sit-and-stand, five times (≥11.20 s)** | N=34 | N=17 |  |
| Age ≥65 years | 13 (38.2%) | 8 (47.1%) | 0.546 |
| ELN 2022 risk |  |  |  |
| Low risk | 8 (23.5%) | 1 (5.9%) | 0.241 |
| Intermediate risk | 18 (52.9% ) | 12 (70.6%) | 0.227 |
| High risk | 8 (23.5%) | 4 (23.5%) | 1.000 |
| Female sex | 9 (26.5%) | 9 (52.9%) | 0.119 |
| Secondary AML | 7 (20.6%) | 3 (17.6%) | 1.000 |
| HCI-CI ≥3 | 5 (14.7%) | 5 (29.4%) | 0.270 |
| Sex mismatch | 15 (44.1%) | 10 (58.8%) | 0.382 |
| ABO match | 23 (67.6%) | 12 (70.6%) | 0.831 |
| RIC | 29 (85.3%) | 16 (94.1%) | 0.650 |
| ATG dosage 5.0 mg/kg | 22 (64.7%) | 15 (88.2%) | 0.102 |
| Donor type |  |  |  |
| MSD | 9 (26.5%) | 1 (5.9%) | 0.135 |
| MUD | 12 (35.3%) | 2 (11.8%) | 0.102 |
| Haploidentical | 13 (38.2%) | 14 (82.4%) | 0.004 |
| **Gait speed (≥4.82 s)** | N=38 | N=13 |  |
| Age ≥65 years | 13 (34.2%) | 8 (61.5%) | 0.109 |
| ELN 2022 risk |  |  |  |
| Low risk | 9 (23.7%) | 0 (0%) | 0.090 |
| Intermediate risk | 22 (57.9%) | 8 (61.5%) | 1.000 |
| High risk | 7 (18.4%) | 5 (38.5%) | 0.254 |
| Female sex | 12 (31.6%) | 6 (46.2%) | 0.502 |
| Secondary AML | 7 (18.4%) | 3 (23.1%) | 0.701 |
| HCI-CI ≥3 | 6 (15.8%) | 4 (30.8%) | 0.253 |
| Sex mismatch | 17 (44.7%) | 8 (61.5%) | 0.296 |
| ABO match | 26 (68.4%) | 9 (69.2%) | 1.000 |
| RIC | 33 (86.8%) | 12 (92.3%) | 1.000 |
| ATG dosage 5.0 mg/kg | 25 (65.8%) | 12 (92.3%) | 0.082 |
| Donor type |  |  |  |
| MSD | 10 (26.3%) | 0 (0%) | 0.052 |
| MUD | 12 (31.6%) | 2 (15.4%) | 0.472 |
| Haploidentical | 16 (42.1%) | 11 (84.6%) | 0.010 |
| **MNA (≤23.5, malnutrition)** | N=48 | N=3 |  |
| Age ≥65 years | 18 (37.5%) | 3 (100%) | 0.064 |
| ELN 2022 risk |  |  |  |
| Low risk | 9 (18.8%) | 0 (0%) | 1.000 |
| Intermediate risk | 27 (56.3%) | 3 (100%) | 0.259 |
| High risk | 12 (25.0%) | 0 (0%) | 1.000 |
| Female sex | 17 (35.4%) | 1 (33.3%) | 1.000 |
| Secondary AML | 10 (20.8%) | 0 (0%) | 1.000 |
| HCI-CI ≥3 | 8 (16.7%) | 2 (66.7%) | 0.094 |
| Sex mismatch | 24 (50.0%) | 1 (33.3%) | 1.000 |
| ABO match | 32 (66.7%) | 3 (100%) | 0.543 |
| RIC | 42 (87.5%) | 3 (100%) | 1.000 |
| ATG dosage 5.0 mg/kg | 34 (70.8%) | 3 (100%) | 0.552 |
| Donor type |  |  |  |
| MSD | 10 (20.8%) | 0 (0%) | 1.000 |
| MUD | 14 (29.2%) | 0 (0%) | 0.552 |
| Haploidentical | 24 (50.0%) | 3 (100%) | 0.238 |
| **OARS (≥18, impaired)** | N=44 | N=7 |  |
| Age ≥65 years | 20 (45.5%) | 1 (14.3%) | 0.217 |
| ELN 2022 risk |  |  |  |
| Low risk | 7 (15.9%) | 2 (28.6%) | 0.592 |
| Intermediate risk | 27 (61.4%) | 3 (42.9%) | 0.427 |
| High risk | 10 (22.7%) | 2 (28.6%) | 0.662 |
| Female sex | 14 (31.8%) | 4 (57.1%) | 0.226 |
| Secondary AML | 9 (20.5%) | 1 (14.3%) | 1.000 |
| HCI-CI ≥3 | 10 (22.7%) | 0 (0%) | 0.320 |
| Sex mismatch | 22 (50.0%) | 3 (42.9%) | 1.000 |
| ABO match | 30 (68.2%) | 5 (71.4%) | 1.000 |
| RIC | 39 (88.6%) | 6 (85.7%) | 1.000 |
| ATG dosage 5.0 mg/kg | 33 (75.0%) | 4 (57.1%) | 0.376 |
| Donor type |  |  |  |
| MSD | 8 (18.2%) | 2 (28.6%) | 0.612 |
| MUD | 11 (25.0%) | 3 (42.9%) | 0.376 |
| Haploidentical | 25 (56.8%) | 2 (28.6%) | 0.232 |
| **MMSE-KC (≤23, impaired)** | N=41 | N=10 |  |
| Age ≥65 years | 15 (36.6%) | 6 (60.0%) | 0.283 |
| ELN 2022 risk |  |  |  |
| Low risk | 8 (19.5%) | 1 (10.0%) | 0.667 |
| Intermediate risk | 22 (53.7%) | 8 (80.0%) | 0.167 |
| High risk | 11 (26.8%) | 1 (10.0%) | 0.417 |
| Female sex | 12 (29.3%) | 6 (60.0%) | 0.137 |
| Secondary AML | 10 (24.4%) | 0 (0%) | 0.178 |
| HCI-CI ≥3 | 7 (17.1%) | 3 (30.0%) | 0.389 |
| Sex mismatch | 20 (48.8%) | 5 (50.0%) | 1.000 |
| ABO match | 31 (75.6%) | 4 (40.0%) | 0.054 |
| RIC | 35 (85.4%) | 10 (100%) | 0.331 |
| ATG dosage 5.0 mg/kg | 30 (73.2%) | 7 (70.0%) | 1.000 |
| Donor type |  |  |  |
| MSD | 9 (22.0%) | 1 (10.0%) | 0.664 |
| MUD | 12 (29.3%) | 2 (20.0%) | 0.707 |
| Haploidentical | 20 (48.8%) | 7 (70.0%) | 0.300 |
| **SGDS-K (≥6, impaired)** | N=46 | N=5 |  |
| Age ≥65 years | 20 (43.5%) | 1 (20.0%) | 0.391 |
| ELN 2022 risk |  |  |  |
| Low risk | 9 (19.6%) | 0 (0%) | 0.571 |
| Intermediate risk | 28 (60.9%) | 2 (40.0%) | 0.637 |
| High risk | 9 (19.6%) | 3 (60.0%) | 0.078 |
| Female sex | 17 (37.0%) | 1 (20.0%) | 0.645 |
| Secondary AML | 10 (21.7%) | 0 (0%) | 0.569 |
| HCI-CI ≥3 | 10 (21.7%) | 0 (0%) | 0.569 |
| Sex mismatch | 24 (52.2%) | 1 (20.0%) | 0.350 |
| ABO match | 31 (67.4%) | 4 (80.0%) | 1.000 |
| RIC | 40 (87.0%) | 5 (100%) | 1.000 |
| ATG dosage 5.0 mg/kg | 33 (71.7%) | 4 (80.0%) | 1.000 |
| Donor type |  |  |  |
| MSD | 10 (21.7%) | 0 (0%) | 0.569 |
| MUD | 13 (28.3%) | 1 (20.0%) | 1.000 |
| Haploidentical | 23 (50.0%) | 4 (80.0%) | 0.354 |
| **NCCN distress thermometer reading (≥3, impaired)** | N=36 | N=15 |  |
| Age ≥65 years | 15 (41.7%) | 6 (40.0%) | 0.912 |
| ELN 2022 risk |  |  |  |
| Low risk | 8 (22.2%) | 1 (6.7%) | 0.251 |
| Intermediate risk | 20 (55.6%) | 10 (66.7%) | 0.463 |
| High risk | 8 (22.2%) | 4 (26.7%) | 0.730 |
| Female sex | 13 (36.1%) | 5 (33.3%) | 0.850 |
| Secondary AML | 7 (19.4%) | 3 (20.0%) | 1.000 |
| HCI-CI ≥3 | 7 (19.4%) | 3 (20.0%) | 1.000 |
| Sex mismatch | 18 (50.0%) | 7 (46.7%) | 0.828 |
| ABO match | 10 (27.8%) | 6 (40.0%) | 0.510 |
| RIC | 33 (91.7%) | 12 (80.0%) | 0.343 |
| ATG dosage 5.0 mg/kg | 27 (75.0%) | 10 (66.7%) | 0.732 |
| Donor type |  |  |  |
| MSD | 7 (19.4%) | 3 (20.0%) | 1.000 |
| MUD | 9 (25.0%) | 5 (33.3%) | 0.732 |
| Haploidentical | 20 (55.6%) | 7 (46.7%) | 0.562 |

ADL, Activities of daily living; AML, acute myeloid leukemia; ATG, anti-thymocyte globulin; ELN, European leukemia network; HCT-CI, hematopoietic cell transplantation-specific comorbidity index; K-IADL, Korean instrumental activities of daily living; K-MBI, Korean version of modified Barthel index; MMSE-KC, mini-mental state examination-the Korean version of CERAD assessment packet; MNA, mini Nutritional Assessment; MSD, matched sibling donor; MUD, matched unrelated donor; NCCN, National Comprehensive Cancer Network; OARS, Older Americans Resources and Services; RIC, reduced-intensity conditioning; SGDS-K, the Korean version of short form geriatric depressive scale; and SPPB, short physical performance battery
