# Supplementary figures and images for: Dynamic changes in physical function during intensive chemotherapy affect transplant outcomes in older adults with AML
Source: Front Oncol. 2023 Nov 7;13:1281782. doi: 10.3389/fonc.2023.1281782 (PMC10661959; doi:10.3389/fonc.2023.1281782)

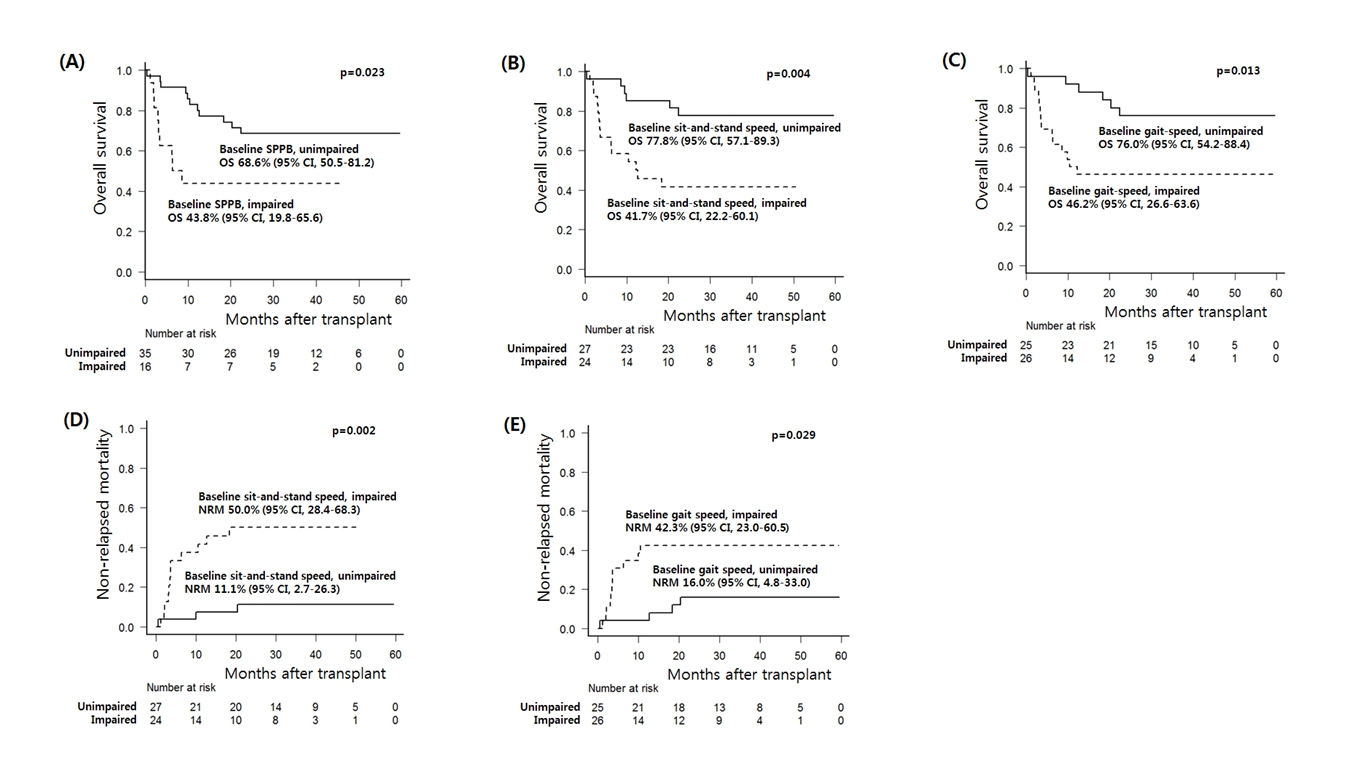

Supplement: Supplementary file 2 [file Image_1.jpeg]

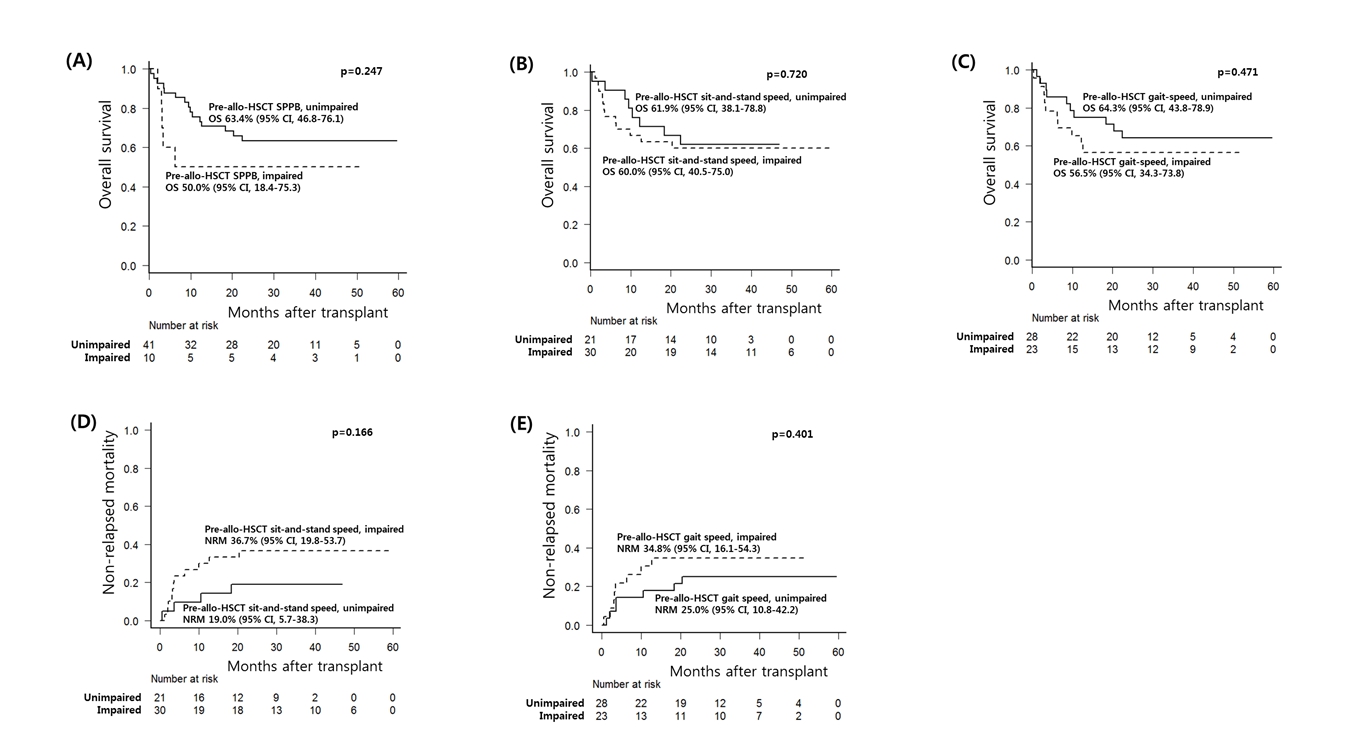

Supplement: Supplementary file 3 [file Image_2.jpeg]

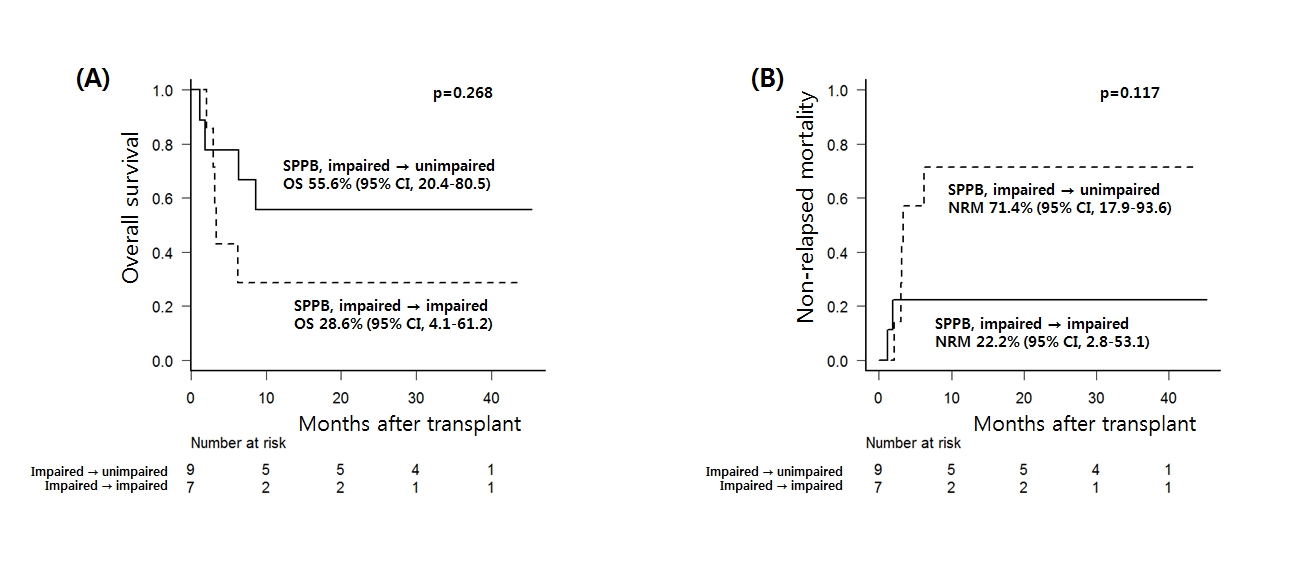

Supplement: Supplementary file 4 [file Image_3.jpeg]
